# Muscle quantitative MRI as a novel biomarker in hereditary transthyretin amyloidosis with polyneuropathy: a cross-sectional study

**DOI:** 10.1007/s00415-022-11336-z

**Published:** 2022-09-06

**Authors:** Elisa Vegezzi, Andrea Cortese, Niels Bergsland, Roberta Mussinelli, Matteo Paoletti, Francesca Solazzo, Riccardo Currò, Lucia Ascagni, Ilaria Callegari, Ilaria Quartesan, Alessandro Lozza, Xeni Deligianni, Francesco Santini, Enrico Marchioni, Giuseppe Cosentino, Enrico Alfonsi, Cristina Tassorelli, Stefano Bastianello, Giampaolo Merlini, Giovanni Palladini, Laura Obici, Anna Pichiecchio

**Affiliations:** 1 Department of Brain and Behavioral Sciences, University of Pavia, Pavia, Italy; 2 Neuroncology and Neuroinflammation Unit, IRCCS Mondino Foundation, Pavia, Italy; 3 Department of Neuromuscular Disease, UCL Queen Square Institute of Neurology and The National Hospital for Neurology and Neurosurgery, London, UK; 4 Department of Neurology, Buffalo Neuroimaging Analysis Center, Jacobs School of Medicine and Biomedical Sciences, University at Buffalo, State University of New York, Buffalo, NY USA; 5 IRCCS Fondazione Don Carlo Gnocchi ONLUS, Milan, Italy; 6 Amyloidosis Research and Treatment Center, Fondazione IRCCS Policlinico San Matteo, University of Pavia, Pavia, Italy; 7 Neuroradiology Department, Advanced Imaging and Radiomics Center, IRCCS Mondino Foundation, Pavia, Italy; 8 Specialization School in Occupational Medicine, University of Pavia, Pavia, Italy; 9 Neuroscience Department, Meyer Children’s University Hospital, University of Florence, Florence, Italy; 10 Department of Biomedicine, University Hospital Basel, University of Basel, Hebelstrasse 20, 4031 Basel, Switzerland; 11 Division of Radiological Physics, Department of Radiology, University Hospital Basel, Basel, Switzerland; 12 Department of Biomedical Engineering, Basel Muscle MRI Group, University of Basel, Allschwil, Switzerland; 13 Translational Neurophysiology Research Unit, IRCCS Mondino Foundation, Pavia, Italy; 14 Headache Science and Neurorehabilitation Center, IRCCS Mondino Foundation, Pavia, Italy; 15 Department of Molecular Medicine, University of Pavia, Pavia, Italy

**Keywords:** Polyneuropathy, ATTR, Magnetic resonance imaging (MRI), Outcome measure, Biomarker

## Abstract

**Background:**

The development of reproducible and sensitive outcome measures has been challenging in hereditary transthyretin (ATTRv) amyloidosis. Recently, quantification of intramuscular fat by magnetic resonance imaging (MRI) has proven as a sensitive marker in patients with other genetic neuropathies. The aim of this study was to investigate the role of muscle quantitative MRI (qMRI) as an outcome measure in ATTRv.

**Methods:**

Calf- and thigh-centered multi-echo T2-weighted spin-echo and gradient-echo sequences were obtained in patients with ATTRv amyloidosis with polyneuropathy (*n* = 24) and healthy controls (*n* = 12). Water T2 (wT2) and fat fraction (FF) were calculated. Neurological assessment was performed in all ATTRv subjects. Quantitative MRI parameters were correlated with clinical and neurophysiological measures of disease severity.

**Results:**

Quantitative imaging revealed significantly higher FF in lower limb muscles in patients with ATTRv amyloidosis compared to controls. In addition, wT2 was significantly higher in ATTRv patients. There was prominent involvement of the posterior compartment of the thighs. Noticeably, FF and wT2 did not exhibit a length-dependent pattern in ATTRv patients. MRI biomarkers correlated with previously validated clinical outcome measures, Polyneuropathy Disability scoring system, Neuropathy Impairment Score (NIS) and NIS-lower limb, and neurophysiological parameters of axonal damage regardless of age, sex, treatment and TTR mutation.

**Conclusions:**

Muscle qMRI revealed significant difference between ATTRv and healthy controls. MRI biomarkers showed high correlation with clinical and neurophysiological measures of disease severity making qMRI as a promising tool to be further investigated in longitudinal studies to assess its role at monitoring onset, progression, and therapy efficacy for future clinical trials on this treatable condition.

**Supplementary Information:**

The online version contains supplementary material available at 10.1007/s00415-022-11336-z.

## Introduction

Hereditary transthyretin (ATTRv; "v" for variant) amyloidosis is a rare systemic disease caused by mutations in the transthyretin (*TTR*) gene. Mutant TTR protein tends to misfold and accumulates as amyloid extracellular fibrils across different tissues and organs, especially peripheral nerves, and heart. Since its identification, more than 150 amyloidogenic mutations have been reported, with a broad phenotypic variability. The most frequent mutation is Val30Met (alternatively named p.Val50Met) which was the first one identified. ATTRv amyloidosis is a progressive, disabling and life-threatening condition with a mean survival of 7–10 years after onset if left untreated [1–3].

Different therapies have been approved for ATTRv amyloidosis so far, including the TTR stabilizer tafamidis and, more recently, the RNAi agent patisiran and the antisense oligonucleotide inotersen [4, 5]. Moreover, other treatment options are underway [6]. Therefore, there is a constant need for sensitive biomarkers to help establishing the disease onset, to track its progression and to monitor the drug efficacy.

Similar to other neuromuscular disorders, the identification of outcome measures has always proved challenging in ATTRv amyloidosis. To date, clinically based scales as Neuropathy Impairment Score (NIS) [7], NIS-lower limb (NIS-LL) [7, 8], NIS + 7 [7, 9–11] and modified NIS + 7 (mNIS + 7) [4, 5, 9, 10] have been used in clinical trials to assess progression and treatment response, however, all them have limitations including inter-rater variability and their dependence on patient’s motivation.

Muscle quantitative MRI (qMRI) has been extensively used as an outcome measure in muscle diseases [12–15]. More recently, the quantification of intramuscular fat substitution (fat fraction, FF), which indirectly reflects axonal degeneration, showed high responsiveness to change over 12 months in patients with genetic neuropathies including Charcot-Marie-Tooth 1A (CMT1A) and hereditary sensory neuropathy type 1 due to SPTLC1 and SPTLC2 mutations [16, 17]. In motoneuron diseases (amyotrophic lateral sclerosis, and spinal bulbar muscular atrophy) qMRI revealed significant fat substitution compared to controls. It also correlated with clinical measures, and identified distinct patterns of muscle involvement [18]. Moreover, STIR and relative T2-weighted signal turned out as objective surrogate markers of muscle denervation, and significantly increased over 12 months [18, 19].

The aim of our cross-sectional study was to assess the role of qMRI of skeletal muscle as an outcome measure in ATTRv amyloidosis with polyneuropathy (ATTRv-PN) and compare it with previously validated and functionally relevant clinical outcomes.

## Materials and methods

### Study design and patient recruitment

We performed a prospective cross-sectional study assessing muscle qMRI of the lower limbs in symptomatic patients diagnosed with ATTRv-PN (*n* = 24) who were enrolled among those who attended the Amyloidosis Research and Treatment Center (IRCCS Fondazione Policlinico S. Matteo) in Pavia (Italy) between September 2017 and August 2018.

ATTRv-PN patients were defined as symptomatic when Polyneuropathy Disability (PND) [20] scored >  = 1.

Healthy controls (HCs), group-matched for age and sex, were also enrolled (*n *= 12). Exclusion criteria for all participants were pregnancy and safety-related MRI contraindications.

### Data acquisition: clinical and functional testing and electrophysiological revision

All patients underwent detailed assessment by E.V. including demographic records, past medical history and full neurologic examination. Patients were rated using PND scoring system [20], NIS [7] and NIS-LL [7, 8]. PND score was graded as follows: PND = 1 (sensory disturbances with preserved walking capability), PND = 2 (sensory-motor symptoms with unassisted gait), PND = 3 (sensory-motor symptoms with assisted gait), and PND = 4 (wheelchair-bound or bedridden). In a subset of *n* = 17 patients who underwent nerve conduction studies (NCSs) at the same time from MRI, compound muscle action potential (CMAP) of peroneal and tibial nerve, and sensory nerve action potential (SNAP) of sural nerve, measured form peak to peak, were reviewed in detail and considered for further analysis. For each patient, the most affected side was considered unless the asymmetry was due to other conditions (e.g., radiculopathy).

### Magnetic resonance imaging

Muscle MRI was performed at IRCCS Mondino Foundation. Participants were examined on a 3 T scanner (MAGNETOM Skyra, Siemens, Erlangen, Germany) lying supine and feet-first The acquisition protocol included calf- and thigh-level centered T1-weigthed, short tau inversion recovery (STIR), a 2D multi-echo T2-weighted spin-echo (SE) (ME-SE) (number of echoes 17, number of slices 7, repetition time (TR) 4100 ms, first echo time (TE) and echo spacing 10.9 ms, bandwidth 250 Hz/px, matrix size 192 × 384, resolution 1.2 × 1.2 mm^2^, slice thickness 10 mm, gap between slices 30 mm), and a 3D gradient-echo (ME-GRE) (number of echoes 6, TR 35 ms, first TE/echo spacing 1.7/1.5 ms, flip angle 7°, bandwidth 1050 Hz/px, matrix size 396 × 432 × 52, resolution 1.0 × 1.0 × 5.0 mm^3^). The sequence had a monopolar readout with interleaved echo spacing (even and odd echoes acquired in subsequent repetitions). Imaging of thigh and calf took approximately 35 and 25 min, respectively. 

### MRI data analysis: muscle quantitative MRI 

A single observer (E.V.) with a 5-year training expertise and blinded to study groups outlined for each participant regions of interest (ROIs) on the 1st echo of the ME-SE sequence at mid-thigh (12 muscles) and mid-calf level (6 muscles) using ITK-SNAP software [21]. ROIs were then transferred to the 1st echo of the ME-GRE acquisition and manually adjusted to ensure proper alignment. All ROIs were verified by two expert neuroradiologists (A.P. and M.P.) with more than 5-year expertise in neuromuscular imaging.

The ME-SE sequence was processed using a bi-component extended phase graph algorithm, implemented in Python, for quantification of water T2 (wT2) [22, 23], using an open-source toolbox [24]. The Fatty Riot algorithm was used offline for the calculation of fat/water images from the ME-GRE acquisition [25, 26] and then FF maps were obtained (FF = F/F + W*100%) from each ROI. Average values of FF and wT2 were calculated for the global ROI and for each muscle at thigh and calf level.

**Fig. 1:**
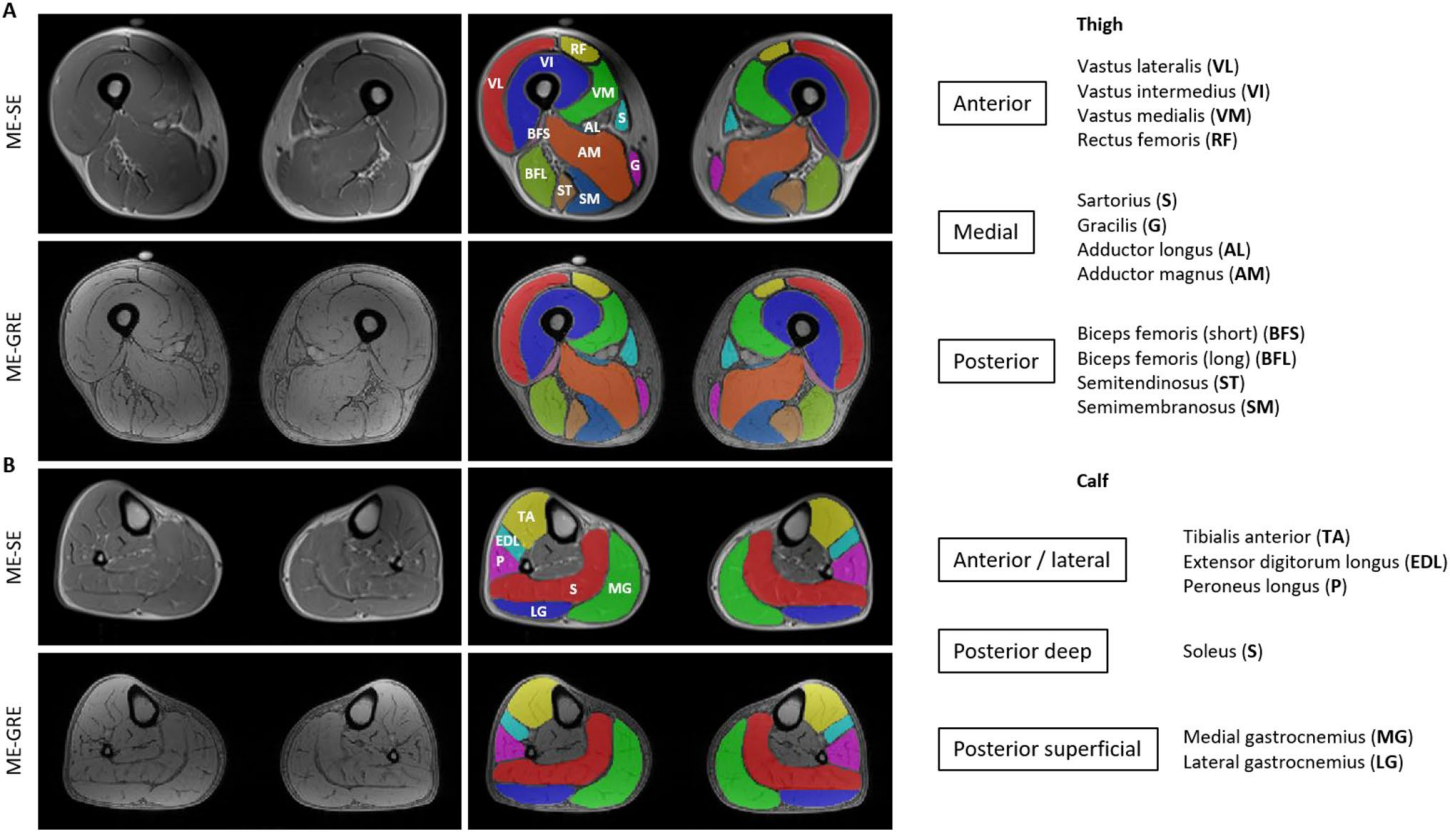
Thigh and calf single muscle ROIs and compartments. Thigh (**A**) and calf (**B**) single muscle ROI of a healthy control superimposed on multi-echo spin-echo (ME-SE) sequence (1st echo) to extract water T2 values and on multi-echo gradient-echo (ME-GRE) sequence (1st echo) to obtain fat fraction maps. Thigh and calf compartments are reported on the right

Figure 1 shows the muscles of the lower limbs which have been assessed. The tibialis posterior (TP) was not evaluated according to the poor quality of visualization at calf MRI.

### Statistical analysis

Statistical analyses were performed with SPSS version 22 (SPSS, Armonk, NY) with a significance α level of 0.05. Quantitative measures are reported as mean ± standard deviation (SD) or median and interquartile range (IQR) as appropriate according to their distribution. For intergroup comparisons two-sample t test and Mann–Whitney U test were applied as appropriate. Correlations of MRI parameters with clinical and electrophysiological measures were investigated with Spearman (*ρ*) or Pearson coefficients as appropriate according to data distribution. Graphics were obtained using Prism-GraphPad version 9.2.0 (332).

## Results

### Participant clinical and demographic records

We enrolled patients with ATTRv-PN (*n* = 24) and healthy controls, group-matched for age and sex (*n* = 12). Seventeen/24 (71%) patients were males, median age at enrollment was 63.5 years (range 42–77), and median disease duration was 5 years (range 4–11).

TTR mutations were: Val30Met (*n* = 6, 25%), Phe64Leu (*n* = 5, 21%), Glu89Gln (*n* = 3, 13%), Tyr78Phe (*n* = 3, 13%), Thr49Ala (*n* = 2, 8%), Ala109Ser (*n* = 2, 8%), Ile68Leu (*n* = 1, 4%), Ser77Tyr (*n* = 1, 4%), Ala49Met (*n* = 1, 4%).

The distribution of PND score was the following: PND = 1 (*n* = 11, 46%), PND = 2 (*n* = 9, 37.5%), PND = 3 (*n* = 3, 12.5%), and PND = 4 (*n* = 1, 4%). Median NIS total and NIS-LL were 25.5 (range 0–170.5) and 14.5 (range 0–88), respectively. Twenty-one/24 (87.5%) were on treatment including tafamidis (*n* = 15, 71%), diflunisal (*n* = 3, 14%), inotersen (*n* = 2, 10%), and patisiran (*n* = 1, 5%). Seventeen/24 (71%) agreed also to undergo NCS evaluation.

Nine/12 (75%) HCs were males and median age at enrollment was 59 years (range 46–68). Demographic and clinical data of the participants are summarized in Table 1 and Supplementary Table 1.

**Table 1:** Demographic and clinical data of ATTRv patients and healthy controls

Demographics and clinical measures	ATTRv patients (*n* = 24)	Control group (*n* = 12)	*p* value
Sex, M/F	17/7	9/3	0.80
Age, y	63.5 (42–77)	59 (46–68)	0.14
Median disease duration (range), y	5 (4–11)	NA	
Treatment, N/tot	21/24	NA	
Tafamidis	15/24		
Diflunisal	3/24		
Inotersen	2/24		
Patisiran	1/24		
Mutation		NA	
Val30Met	6/24		
Phe64Leu	5/24		
Glu89Gln	3/24		
Tyr78Phe	3/24		
Thr49Ala	2/24		
Ala109Ser	2/24		
Ile68Leu	1/24		
Ser77Tyr	1/24		
Ala49Met	1/24		
PND score		NA	
0	NA		
1–2	20/24		
3–4	4/24		
Median NIS (range)	25.5 (0–170.5)	NA	
Median NIS-LL (range)	14.5 (0–80)	NA	

### Muscle fat fraction and water T2 distinguish patients with ATTRv amyloidosis from healthy controls

We found that FF was significantly higher in ATTRv patients compared to healthy controls at thigh (ATTRv vs controls: median 9.8%, IQR 7.3% vs median 6.5%, IQR 2.5%; *p* = 0.002) and calf level (ATTRv vs controls: median 9.9%, IQR 6% vs median 7.1%, IQR 3.1%; *p* = 0.017).

**Fig. 2:**
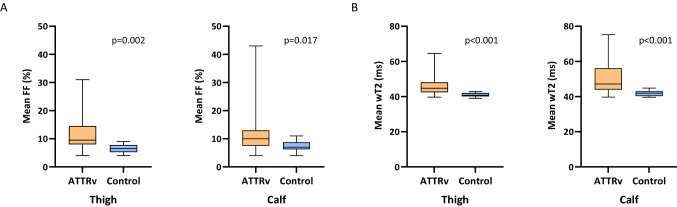
Muscle quantitative MRI imaging: thigh and calf compartments in ATTRv patients and healthy controls. Overall thigh and calf fat fraction (FF) and water T2 (wT2) are significantly higher in ATTRv compared to healthy controls. Boxes represent median and IQR and whiskers show range

Similarly, wT2 was significantly higher in ATTRv patients compared to healthy controls, both in the thighs (ATTRv vs controls: median 44.8 ms, IQR 5.6 ms vs median 40.8 ms, IQR 1.8 ms; *p* < 0.001) and in the calves (ATTRv vs controls: median 47.2 ms, IQR 12.2 ms vs median 42 ms, IQR 2.7 ms; *p* < 0.001) (Table 2; Fig. 2).

**Table 2:** Fat fraction (FF) and water T2 (wT2) of ATTRv patients and healthy controls

Quantitative imaging measures	ATTRv patients (*n* = 24)	Healthy controls (*n* = 12)	*p* value
MRI, thigh level			
FF, %	9.8 (4.1–31.3)	6.5 (4.2–9.0)	0.002
wT2, ms	44.8 (39.7–64.6)	40.8 (39.0–42.8)	< 0.001
MRI, calf level			
FF, %	9.9 (4.0–42.8)	7.1 (4.1–11.5)	0.017
wT2, ms	47.2 (39.7–75.2)	42.0 (39.6–44.8)	< 0.001

Data presented as median (range) as appropriate to distribution; *p* values calculated with Mann–Whitney U test

No significant difference was seen between patients carrying Val30Met (p.Val50Met) and other mutations (data not shown).

### Quantitative MRI parameters correlate with clinical outcomes in ATTRv amyloidosis

We next assessed the role of qMRI as a biomarker of disease severity by looking at the correlation between qMRI measures and previously validated scales of disability (PND score) and neurologic impairment (NIS and NIS-LL).

**Fig. 3:**
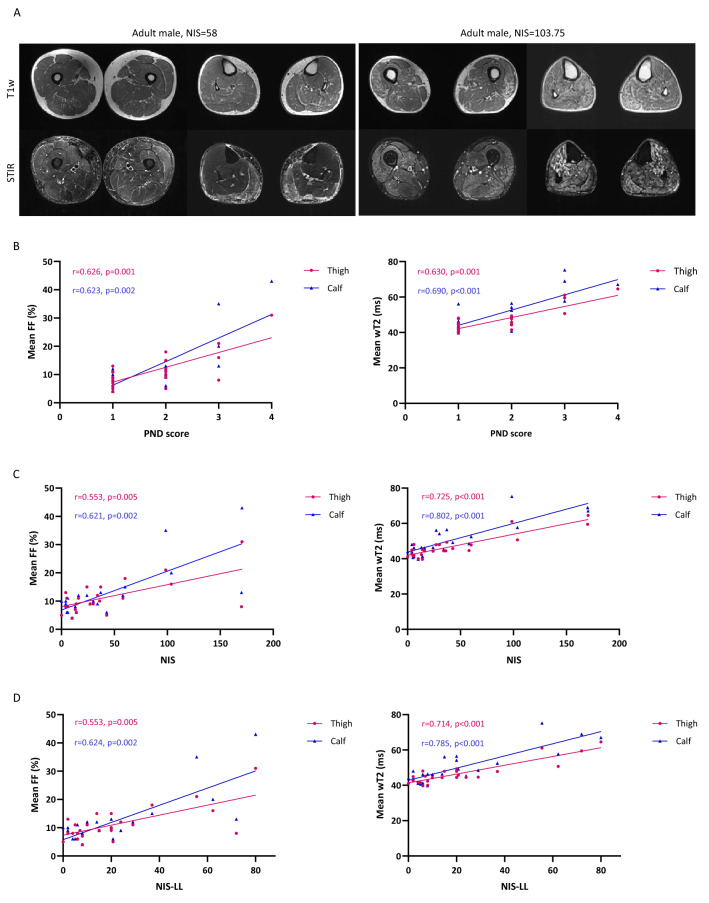
Correlation of muscle quantitative MRI measures with functional rating scales in ATTRv amyloidosis. Representative examples of T1-weighted (T1w) and short tau inversion recovery (STIR) sequences of the thighs and calves of two patients affected by ATTRv amyloidosis with moderate (left) and severe (right) disease. The patient with greater disability (right) has more prominent fat infiltration and a higher water T2 content (**A**). Moderate to strong positive correlation was observed between mean fat fraction (FF) and water T2 (wT2) at thigh (purple) and calf (blue) level and Polyneuropathy Disability (PND) score (**B**), Neuropathy Impairment Score (NIS) (**C**), and NIS-lower limb (NIS-LL) (**D**)

Thigh and calf FF correlated well with PND score (thigh: *r* = 0.626, *p* = 0.001; calf: *r* = 0.623, *p* = 0.002), NIS (thigh: *r* = 0.553, *p* = 0.005; calf: *r* = 0.621, *p* = 0.002), and NIS-LL (thigh: *r* = 0.553, *p* = 0.005; calf: *r* = 0.624, *p* = 0.002). Similarly, water T2 significantly correlated with PND score (thigh: *r* = 0.630, *p* = 0.001; calf: *r* = 0.690, *p* < 0.001), NIS (thigh: *r* = 0.725, *p* < 0.001; calf: *r* = 0.802, *p* < 0.001), and NIS-LL (thigh: *r* = 0.714, *p* < 0.001; calf: *r* = 0.785, *p* < 0.001) (Fig. 3). These positive associations were independent from sex, age, treatment and mutation in a multivariable linear regression model (Supplementary Table 2).

### Quantitative MRI parameters correlate with NCS measures in ATTRv amyloidosis

**Fig. 4:**
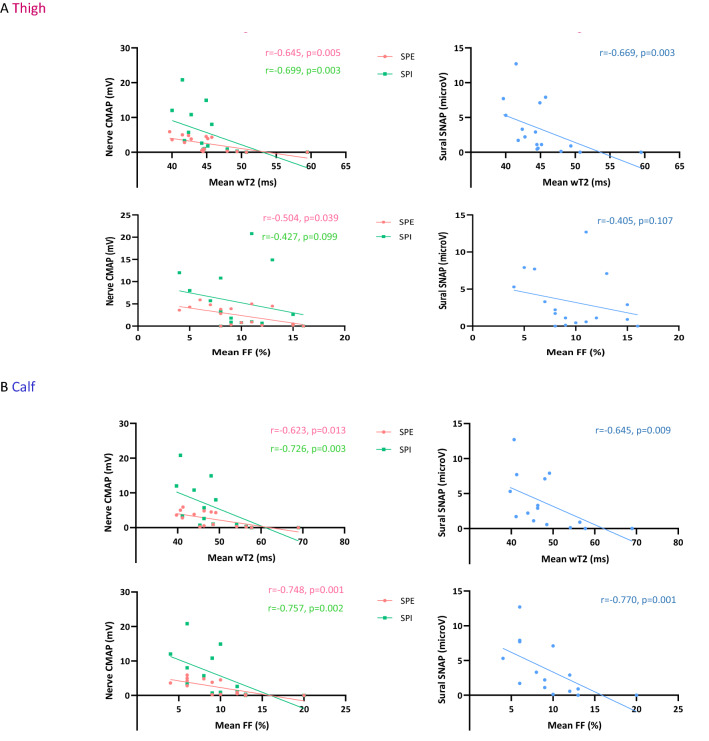
Correlation of muscle quantitative MRI measures with NCS parameters in ATTRv amyloidosis. Negative correlation between NCS parameters namely peroneal nerve compound muscle action potential (CMAP) (pink), tibial nerve CMAP (green), and sural nerve sensory nerve action potential (SNAP) (light-blue) and water T2 (wT2) and fat fraction (FF) at thigh (**A**) and calf (**B**) level

Quantitative MRI parameters also showed significant correlation with several neurophysiological parameters. In particular, FF and wT2 negatively correlated with peroneal nerve CMAP (thigh: FF: *r* = − 0.504, *p* = 0.039; wT2: *r* = − 0.645, *p* = 0.005; calf: FF: *r* = − 0.748, *p* = 0.001; wT2: *r* = − 0.623, *p* = 0.013), tibial nerve CMAP (thigh: wT2: *r* = − 0.699, *p* = 0.003; calf: FF: *r* = − 0.757, *p* = 0.002; wT2: *r* = − 0.726, *p* = 0.003), and sural nerve SNAP (thigh: wT2: *r* = − 0.669, *p* = 0.003; calf: FF: *r* = − 0.770, *p* = 0.001; wT2: *r* = − 0.645, *p* = 0.009) amplitudes (Fig. 4).

Interestingly, in 7/17 (42%), ATTRv patients with unexcitable or severely reduced motor and sensory action potentials in the lower limbs (peroneal and tibial CMAP <  = 1 mV, sural SNAP <  = 1 microV) qMRI showed changes ranging from + 8% to + 16% of FF at thighs and + 9% to + 20% at calves, compared to an average in controls of 6.5% and 7.1%, respectively, correlating with clinical severity.

### Muscle fat fraction and water T2 do not exhibit length-dependent changes in ATTRv amyloidosis

ATTRv amyloidosis typically presents with a length-dependent pattern of weakness, namely lower limbs are more affected than upper limbs and distal limb segments are more affected than proximal ones [27].

In our cohort, calf muscles were significantly weaker compared to thigh muscles as measured by NIS-LL (NIS-LL score at calf vs thigh: median 7, IQR 28.5 vs median 0, IQR 6; *p* = 0.023).

**Fig. 5:**
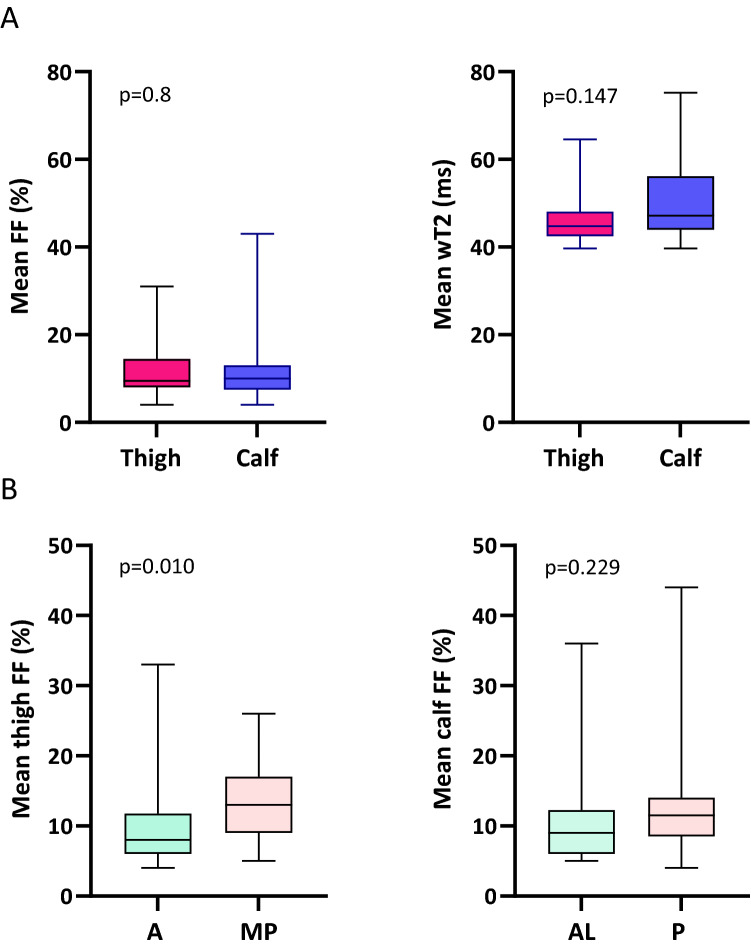
Muscle quantitative MRI imaging: thigh and calf compartments in ATTRv amyloidosis. Fat fraction (FF) and water T2 (wT2) were not significantly different between thigh and calf level (**A**). Fat substitution prevailed in the medio-posterior thigh compartment compared to the anterior region while no difference was seen at calf between the antero-lateral and posterior region (**B**)

Despite these clinical findings, thighs showed a similar degree of fat replacement compared to calves (thighs median FF 9.8%, IQR 7.3% vs calves median FF 9.9%, IQR 6.0%; *p* = 0.8). Similarly, no significant difference was appreciated between wT2 at thigh and calf level (thighs median wT2 44.8, IQR 5.6 vs calves median wT2 47.2, IQR 12.2; *p* = 0.147) (Fig. 5).

### Pattern of fat infiltration: posterior thigh involvement in ATTRv amyloidosis

Quantitative MRI identified a prominent involvement of the medio-posterior compartment of the thighs with a relative sparing of the quadriceps (median 12.8%, IQR 8.6% vs median 7.7%, IQR 5.5%; *p* = 0.010), despite the similar involvement at clinical examination (NIS-LL score anterior vs posterior region at thigh: median 0, IQR 2 vs median 0, IQR 3; *p* = 0.963).

At calf level, no significant difference in FF between different compartments was appreciated (deep posterior vs superficial posterior: *p* = 0.925; deep posterior vs antero-lateral: *p* = 0.336; superficial posterior vs antero-lateral: *p* = 0.250; posterior vs antero-lateral: *p* = 0.229) (Fig. 5).

## Discussion

In this study, we assessed the role of muscle qMRI as a novel outcome measure in a cohort of ATTRv patients with polyneuropathy. We showed that qMRI reveals significant difference between ATTRv patients and healthy controls and strongly correlates with previously validated clinical measures.

ATTRv amyloidosis is a progressive and highly debilitating hereditary disease, which is fatal within a decade without treatment [1–3]. Early diagnosis is key to promptly start an appropriate anti-amyloidogenic treatment. Therefore, there is a need for reliable and objective measures to establish the disease onset, track its progression and monitor the response to treatments.

To date, most outcome measures used in ATTRv-PN are based on clinical examination and functional impairment. In particular, NIS, along with its subset NIS-LL, which was first designed to grade neurological impairment in diabetic neuropathy [28], has become the most used outcome measure in different clinical trials and observational studies in ATTRv amyloidosis [7, 8, 29]. However, it does not encompass the autonomic and cardiac involvement of the disease. Also, even if performed by expert and preliminarily-trained clinicians, NIS and NIS-LL are limited by intra- and inter-rater variability [4, 30, 31] along with patient’s motivation. To better capture the multisystem involvement in ATTRv amyloidosis and reduce its variability [32], novel compound scales, NIS + 7 and mNIS + 7, have been developed [7, 9–11]. Although NIS + 7 and mNIS + 7 provide a thorough evaluation of ATTRv amyloidosis, they are time-consuming and require a bespoke setting and specific training of the examiners [33].

Quantitative MRI may represent an attractive option to overcome shortcomings in clinical examination as it is relatively rapid, with high inter- and intra-operator reproducibility of manual muscle segmentation [21] and analysis can also be automated in all ATTRv patients. In particular, previous studies have shown that muscle quantitative MRI has a very good reproducibility with an interclass correlation coefficient > 0.9 both for inter- and intra-rater agreement [34]. The future implementation of robust machine learning algorithms for automatic segmentation of muscles may be the key to overcome the limitations related to manual segmentation [35, 36].

Recently, MRI neurography of sciatic and sural nerve was shown to be able to accurately distinguish patients with ATTRv amyloidosis from controls and, importantly, to detect subclinical and early nerve lesions in asymptomatic carriers [37, 38]. Similarly, magnetization transfer ratio of the sciatic nerve showed promising results as it differentiated both symptomatic ATTRv patients and asymptomatic carriers from healthy controls and correlated with electrophysiology [39].

Muscle qMRI has been extensively applied as disease biomarker for the study of muscle dystrophies and other myopathies, including Duchenne muscular dystrophy, where both fat fraction and water T2 content provided sensitive noninvasive measures of disease progression over time [12]. More recently, quantification of intramuscular fat showed high responsiveness to change over 12-month time in genetic neuropathies, and currently represents the most sensitive outcome measure for the assessment of the slow progression of CMT1A [16].

Prompted by these encouraging results, we decided to assess the role of muscle qMRI in ATTRv amyloidosis with polyneuropathy. We evaluated both acute (tissue water content as expressed by T2 signal) and chronic (fat replacement as expressed by FF) changes in the lower limb muscles. We found that water T2 and FF were significantly higher in ATTRv patients compared to healthy controls and were able to differentiate the two groups. Their increase might be explained by axonal damage due to the underlying neuropathy resulting in acute (as defined by water T2) and chronic (as defined by FF) denervation. More important, we observed a moderate to strong positive correlation between muscle qMRI parameters (mean FF and wT2) at both thigh and calf level and patients’ functionally relevant clinical measures (PND score, NIS, and NIS-LL scales). Therefore, muscle qMRI represents a reliable surrogate measure of disease severity in ATTRv amyloidosis, which is independent of participant’s effort and with a high intra-rater agreement [21]. In addition, qMRI may help clinicians to monitor disease progression in ATTRv patients in more advanced stages of the disease, when widespread reduction or unexcitability of motor and sensory action potentials limits the role of neurophysiology in assessing progression and response to treatment.

Interestingly, in ATTRv, qMRI showed a similar degree of fat replacement and water content of thigh and calf muscles, which was unexpected considering the length-dependent involvement of lower limb muscles at examination. This finding differed from previous studies on slowly progressive neuropathies, CMT1A and hereditary neuropathy with liability to pressure palsies, where qMRI showed a higher degree of fat substitution at calves [40, 41]. However, it is worth noting that previous pathological studies have shown in ATTRv amyloidosis conspicuous amyloid deposition in dorsal roots and sympathetic ganglia [42]. Also, previous MR neurography studies have detected in ATTRv carriers the presence of early and prominent changes of the proximal nerve tracts compared to distal ones [37], which is in agreement with our finding of significant fat replacement of proximal muscles of the lower limbs.

Finally, skeletal muscle MRI showed a characteristic pattern in ATTRv patients with a preferential involvement of posterior muscles and a relative sparing of quadriceps in the thighs, while in the calves, all muscles appeared to be similarly involved. This pattern has not been previously described in other acquired and genetic neuropathies. Indeed, patients affected by chronic inflammatory demyelinating polyneuropathy had fat infiltration both at biceps femoris and quadriceps [43] while in CMT1A, a predominant degeneration of antero-lateral compartments of calves was reported [40]. Therefore, although this observation warrants further confirmation in larger cohorts, it may be a clue to suspect ATTRv amyloidosis in patients with unexplained axonal neuropathy and, maybe in the future, to help differentiating ATTRv amyloidosis from other genetic and acquired neuropathies.

Our study has some limitations. First, we recruited mostly ATTRv patients with mild and moderate neuropathy, while the more advanced stages of the disease were underrepresented. Second, a muscle biopsy was not performed in any case. Therefore, we cannot rule out the presence of a coexisting myopathy. However, needle EMG was available for *n* = 17 patients and did not show myopathic changes in any of them. Also, amyloid myopathy is infrequent in previous case series [44]. Third, although the time required for muscle MRI was limited to 1 hour and was mostly well tolerated by all subjects, patients with more advanced neuropathy showed lower tolerance of a prolonged supine position, which partly explains their lower recruitment. However, in general, good collaboration from the patient was fundamental to obtain images suitable to quantitative analysis. Hopefully, in the near future compressed sensing and parallel imaging will allow to shorten the acquisition time making this evaluation easier to perform.

Longitudinal studies are warranted to assess the role of qMRI as noninvasive, objective, and sensitive biomarker for the diagnosis and monitoring over time of ATTRv-PN patients, especially in presymptomatic and early symptomatic stages of the disease.

## Supplementary Information

Below is the link to the electronic supplementary material.

Supplementary file1 (DOCX 22 KB)

## Data Availability

Anonymized data not published within this article are available in the Zenodo repository (DOI:10.5281/zenodo.7030896).

## Abbreviations

ATTRv: Hereditary transthyretin amyloidosis ("v" for variant)
TTR: Transthyretin
HC: Healthy control
CMT1A: Charcot-Marie-Tooth 1A
qMRI: Quantitative MRI
FF: Fat fraction
WT2: Water T2
NIS: Neuropathy Impairment Score
NIS-LL: Neuropathy Impairment Score-lower limb
PND: Polyneuropathy disability
NCS: Nerve conduction study
CMAP: Compound muscle action potential
SNAP: Sensory nerve action potential
ME-SE: Multi-echo spin-echo
ME-GRE: Multi-echo gradient-echo
RF: Rectus femoris
VL: Vastus lateralis
VI: Vastus intermedius
VM: Vastus medialis
AM: Adductor magnus
AL: Adductor longus
S: Sartorius
G: Gracilis
BFL: Biceps femoris long head
BFS: Biceps femoris short head
ST: Semitendinosus
SM: Semimembranosus
TA: Tibialis anterior
EDL: Extensor digitorum longus
PL: Peroneus longus
LG: Gastrocnemius lateralis
MG: Gastrocnemius medialis
S: Soleus
TP: Tibialis posterior
MRN: MRI neurography
MTR: Magnetization transfer ratio
