# Supplementary material for: Muscle quantitative MRI as a novel biomarker in hereditary transthyretin amyloidosis with polyneuropathy: a cross-sectional study
Source: J Neurol. 2022 Sep 6;270(1):328–39. doi: 10.1007/s00415-022-11336-z (PMC9813036; doi:10.1007/s00415-022-11336-z)
Supplement: Supplementary file 1 — Supplementary file1 (DOCX 22 KB) [file 415_2022_11336_MOESM1_ESM.docx]

**Supplementary material**

**Supplementary Table 1** Clinical data of ATTRv patients

| **ATTRv patient** | **Age, y/sex** | **Mutation** | **Disease duration, y** | **Treatment** | **PND score** | **NIS** | **NIS-LL** |
| --- | --- | --- | --- | --- | --- | --- | --- |
| 01 | 77/M | Phe64Leu | 5 | Diflunisal | 4 | 170.5 | 80 |
| 02 | 58/F | Glu89Gln | 7 | Tafamidis | 2 | 5 | 5 |
| 03 | 57/M | Val30Met | 10 | - | 1 | 14 | 6 |
| 04 | 68/M | Ala109Ser | 11 | Inotersen | 3 | 170 | 72 |
| 05 | 71/M | Tyr78Phe | 3 | Tafamidis | 1 | 14 | 7 |
| 06 | 71/M | Ile68Leu | 3 | Tafamidis | 1 | 6 | 6 |
| 07 | 57/M | Phe64Leu | 3 | Tafamidis | 2 | 58 | 29 |
| 08 | 73/F | Ser77Tyr | 5 | Tafamidis | 2 | 30 | 20 |
| 09 | 55/M | Glu89Gln | 6 | Inotersen | 2 | 37 | 20 |
| 10 | 46/M | Ala49Met | 4 | Tafamidis | 2 | 24 | 14 |
| 11 | 62/F | Tyr78Phe | 8 | Tafamidis | 1 | 6 | 4 |
| 12 | 68/M | Tyr78Phe | 8 | Tafamidis | 1 | 27 | 15 |
| 13 | 63/M | Val30Met | 4 | Tafamidis | 2 | 36 | 20 |
| 14 | 64/M | Val30Met | 4 | Tafamidis | 1 | 4 | 2 |
| 15 | 64/F | Phe64Leu | 7 | Tafamidis | 1 | 10 | 8 |
| 16 | 61/M | Val30Met | 4 | Tafamidis | 1 | 13 | 8 |
| 17 | 42/F | Val30Met | 9 | - | 1 | 0 | 0 |
| 18 | 65/M | Phe64Leu | 2 | Tafamidis | 2 | 42.75 | 20.75 |
| 19 | 75/M | Ala109Ser | 8 | Patisiran | 2 | 60 | 37 |
| 20 | 45/M | Thr49Ala | 11 | Diflunisal | 3 | 103.75 | 62.25 |
| 21 | 49/F | Thr49Ala | 5 | Tafamidis | 2 | 34 | 24 |
| 22 | 72/M | Phe64Leu | 4 | - | 1 | 16 | 10 |
| 23 | 60/F | Glu89Gln | 3 | Tafamidis | 1 | 4 | 2 |
| 24 | 69/M | Val30Met | 4 | Diflunisal | 3 | 98.5 | 55.5 |

**Supplementary Table 2** Multivariate linear regression model

|  | *NIS | NIS-LL | PND score |
| --- | --- | --- | --- |
| †Age  Sex  Treatment  Mutation  Thigh FF | β=0.047, *p*=0.802  -0.238, 0.227  0.069, 0.722  0.076, 0.698  0.525, **0.012** | 0.004, 0.984  -0.218, 0.240  0.099, 0.591  0.069, 0.711  0.596, **0.004** | -0.119, 0.468  -0.089, 0.594  0.180, 0.291  0.022, 0.896  0.697, <**0.001** |
| †Age  Sex  Treatment  Mutation  Calf FF | -0.025,0.880  -0.202, 0.234  0.078, 0.647  0.213, 0.223  0.683, **0.001** | -0.071, 0.234  -0.186, 0.227  0.111, 0.473  0.222, 0.166  0.732, <**0.001** | -0.201, 0.165  -0.068, 0.629  0.184, 0.214  0.243, 0.110  0.802, <**0.001** |
| †Age  Sex  Treatment  Mutation  Thigh wT2 | -0.169, 0.148  -0.091, 0.427  -0.037, 0.742  0.086, 0.445  0.925, <**0.001** | -0.208, 0.078  -0.087, 0.444  0.003, 0.980  0.087, 0.438  0.926, <**0.001** | -0.319, 0.023  0.009, 0.947  0.106, 0.417  0.057, 0.660  0.915, <**0.001** |
| †Age  Sex  Treatment  Mutation  Calf wT2 | -0.071, 0.610  -0.095, 0.609  -0.056, 0.702  0.206, 0.161  0.840, <**0.001** | -0.110, 0.409  -0.089, 0.519  -0.020, 0.887  0.211, 0.135  0.856, <**0.001** | -0.218, 0.170  -0.001, 0.994  0.070, 0.668  0.222, 0.174  0.828, <**0.001** |

*Dependent variable

†Independent variable; standardized regression coefficients (β) and p values (*p*) are shown

Level of significance (α) of 0.05
